# Validation and benchmarks for the Copenhagen Psychosocial Questionnaire (COPSOQ III) in an Australian working population sample

**DOI:** 10.1186/s12889-025-21845-x

**Published:** 2025-03-01

**Authors:** Mark Rahimi, Ben Arnold, Anthony D. LaMontagne, Philip Riley

**Affiliations:** 1 School of Education, Faculty of Arts & Education, Deakin University, Melbourne, Australia; 2 School of Health & Social Development, Faculty of Health, Deakin University, Melbourne, Australia

**Keywords:** Psychosocial risk assessment, Psychosocial risk management, Benchmark, Organisational and social work environment, Psychometric evaluation, Occupational health, COPSOQ

## Abstract

**Background:**

This study presents an analysis of the reliability and validity of the long version of the Copenhagen Psychosocial Questionnaire (COPSOQ III) in Australia and establishes benchmarks for key dimensions of psychosocial work environment.

**Methods:**

A random sample was sourced from a panel of 2,446 working Australians aged 18–79. The study establishes population benchmarks for key measures of the psychosocial work environment and employee mental health, and assesses variation by gender, employment status, work sector, professional status, and occupational classification. Validation entailed an assessment of internal consistency reliability of the measures, followed by a preliminary Exploratory Factor Analysis (EFA) to initially examine construct validity of the instrument, and continued with a Confirmatory Factor Analysis (CFA) to validate the scales. Working population mean scores for the key dimensions of COPSOQ-III were calculated and presented as benchmarks for use in workplace risk assessment and management.

**Results:**

Except for one measure, the analysis revealed strong reliability coefficients for 31 measures with more than two items (> 0.7). Only one measure for ‘demand for hiding emotions’ with more than two items had a reliability coefficient of 0.663. The EFA unveiled a four-factor structure in psychosocial working conditions, and a two-factor model in factors related to the effects. The CFA involved item-level analysis, leading to the removal of seven items to improve model fit.

**Conclusion:**

The present study provides a high-level validation of the long version of COPSOQ, and thus supports its use as an instrument for research as well as psychosocial risk assessment and management in Australia.

## Background

Increasing recognition of the importance of the workplace in worker’s psychological health has driven a focus on managing psychosocial risks and promoting worker health and wellbeing [1]. In many countries, such as Australia, occupational health and safety (OHS) legislation is evolving to require employers to manage psychosocial risks and implement control measures to eliminate or minimise them as far as reasonably practicable [2]. Consequently, the valid and reliable assessment of the psychosocial work environment is crucial for occupational safety and health management and the promotion of worker health [2].

Evidence strongly indicates that the psychosocial work environment significantly impacts worker health [1, 3]. Psychosocial hazards or risks are elements in the design or management of work that heighten the risk of work-related stress [1]. In Australia, regulators have identified common psychosocial hazards, including job demands, low job control, poor support, lack of role clarity, inadequate organisational change management, insufficient recognition and reward, poor organisational justice, exposure to traumatic events, and workplace violence. They provide practical guidance and frameworks to help organisations identify and manage these risks.

Researchers and policymakers are increasingly focused on identifying and mitigating key predictors of psychological health and safety at work [4, 5]. The People at Work Survey, developed in 2008, is a free psychosocial risk assessment tool for Australian workplaces that supports employers to comply with regulation and address common workplace risks [4].

COPSOQ, developed in Denmark in 2005, offers a broader, more research-oriented approach. COPSOQ assesses a wide range of psychosocial work environment factors and outcomes, making it suitable for both workplace assessments and advanced research studies [6]. As such, COPSOQ aligns with the psychosocial hazards identified by Australian regulators and supports researchers and practitioners to assess these risks. COPSOQ has been validated in 18 countries and the results from COPSOQ surveys have been extensively published in peer-reviewed journals [7–9]. Several other instruments are available to measure psychosocial factors at work, including the Danish Psychosocial Work Environment Questionnaire (DPQ) [10] and the General Nordic Questionnaire for psychological and social factors at work (QPSNordic) [11]. However, the COPSOQ was selected for this study due to its comprehensive coverage of psychosocial factors, international validation and the fact it has undergone multiple iterations to enhance its robustness and applicability.

COPSOQ’s approach to assessing the psychosocial work environment seeks to balance the need for international compatibility with the specific nuances of local work contexts. COPSOQ’s guidelines recommend that national teams adapt the questionnaire to their country by including a set of essential or “core,” questions, in addition to a range of nationally pertinent scales from the medium and long versions of COPSOQ. National teams should validate the COPSOQ questionnaire and establish reference population benchmarks. Sample means from an organisation or sector are then compared to these benchmarks for the general working population to assess risk levels. Benchmarks based on representative data from the national working population are therefore essential for assessing psychosocial work environments and managing risks [7].

The COPSOQ has been validated in different European countries, including Germany [12], Sweden [13], Spain [14], Denmark [15] and France [16]. However, its only English-language validation with a general working population has occurred in Canada [17]. In Australia, the validation of COPSOQ is limited to the occupation of school leadership [9], and no national validation of COPSOQ to date has included the full range of scales in the long version of COPSOQ III. Unlike the standard approach of creating a tailored national version, our study aimed to validate all COPSOQ measures with a national working population, ensuring the suitability of the full range of measures for the Australian workforce.

This paper presents the validation of the full length, third version of COPSOQ III in an Australian sample. The primary objectives of this study were to:

1. Assess the reliability and validity of the COPSOQ-III instrument and associated measures for psychosocial risk assessment and research within the Australian working population.
2. Establish benchmarks to aid in research and the assessment of the psychosocial work environment in the Australian adult working population.
3. Assess sub-population differences in psychosocial work environments.

## Methods

### Variables

The long version of the COPSOQ III questionnaire comprises 141 items covering 46 dimensions [8]. Participants typically respond using a five-point scale, which varies depending on the question, ranging from ‘always’ to ‘never’ or ‘to a very large extent’ to ‘to a very small extent’. Different reference periods are used for different sections of the questionnaire; for example, most questions on job demands and job content scales refer to the present, while questions about workplace violence refer to experiences over the past 12 months. In addition to the COPSOQ scales, we included 21 questions related to the work situation and personal characteristics of participants.

### Data collection and sample

An invitation to participate in the study was sent via Qualtrics to 9,339 randomly selected respondents from paid panels. Recipients received an email with a secure survey link, which 5,253 recipients opened. Screening criteria included verification of employment status, age (18–79), and residency in Australia. Eligible participants were those currently in employed—full-time, part-time, casual or self-employed/business owners—and quota sampling by sector was conducted based on the primary industry divisions and the state or territory of residence.

Following the eligibility and industry sector quota-based screening, a total of 2,671 participants proceeded with the survey. After data collection, a rigorous data cleaning process resulted in the exclusion of 225 responses due to various factors including incomplete responses, anomalously rapid completion times, and duplicate entries. This checking process assured the risk of presence of bots or artificial data in the final dataset. The final dataset comprised 2,446 responses for COPSOQ III questions, equating to a response rate of 26.2%.

The latest available data on the Australian labour force were extracted from multiple datasets of the Australian Bureau of Statistics (ABS) [18–22] to identify the key characteristics of the workforce. Direct comparisons with ABS working population statistics are challenging because respondents may report their employment type differently from how it is classified by the ABS. Nevertheless, our sample appears to over-represent permanent/on-going workers (usually closer to 50% in working population estimates) and full-time (FT) contract workers (~ 3%) and under-represent casual workers (~ 20%) [23]. Additionally, the conflation of various forms of self-employment complicates direct comparisons with standard ABS classifications. To assess the representativeness of the sample, key attributes of the reference population, such as age, gender, state/territory, geolocation, employment basis, industry, and sector, were compared to the sample population (see Tables 1 and 2 in Appendix).

Table 1 in Appendix provides a brief demographic comparison between the survey sample and the overall Australian workforce. A higher proportion in our sample were female compared to national averages. The age distribution was closely aligned with the national workforce average. State-wise, the sample generally reflects the workforce distribution across Australia, with some minor discrepancies. Occupational sectors in the sample vary slightly from national trends, with notable differences in sectors like Construction (lower) and Public Administration and Safety (lower). The sample has a greater proportion of full-time workers than the national workforce. Additionally, there was a higher representation of public sector employees in the sample. Overall, the sample was reasonably representative of the Australian workforce, though with some variations in certain demographics.

Other demographics variables are listed in Table 2 of the Appendix. The majority of the sample resided in metropolitan areas, with a smaller representation across inner-regional to very remote locations. Participants were required to be currently employed in Australia and possess appropriate work rights. Most participants were Australian citizens (89.4%) or permanent residents (7.8%), with a small proportion on temporary work visas (2.7%). Additionally, 4.1% of respondents identified as having Aboriginal or Torres Strait Islander heritage. While English was the primary language for most participants, a notable proportion were bilingual or multilingual. The sample included a range of company sizes, from large to small, ensuring a balanced representation across different organisation sizes. Detailed data were also collected on key occupational aspects, including total years of work experience, years of experience in the current job type and contract types.

### Occupational classification

Following the Australian Bureau of Statistics 2022 guidelines, key demographic variables were used to classify participants according to the Australian and New Zealand Standard Classification of Occupations (ANZSCO) 1-digit major occupational categories [24]. Participants were categorised based on factors such as employment type, workplace size, role tenure, qualifications, and industry specifics. This ensured alignment with the 1-digit occupational skill levels, reflecting their job and workplace context. In total, 2,309 observations (94.4% of the total) were mapped to the main one-digit ANZSCO occupational categories.

In the second stage, a binary classification of job type into ‘blue-collar’ or ‘white-collar’ categories was derived from the ANZSCO classifications. Roles such as Managers, Professionals, Technicians and Trades Workers, Clerical and Administrative Workers, and Community and Personal Service Workers were designated as “White Collar” due to their office-based environment, requirements for higher levels of education, and lower physical labour demands. Conversely, roles such as Labourers, Machinery Operators and Drivers, and some Skilled Agricultural Workers, were classified as “Blue Collar” due to their physical nature and association with manual work, trades or the operation of machinery.

The distribution of the sample by occupational category is presented in Table 3 in the Appendix. This table indicates that the sample broadly represents major occupational categories and job classes in Australia. However, there is a slight over-representation of blue-collar workers in the sample (40.9%) compared to the proportion in the Australian working population (31%).

## Analysis

All COPSOQ-III measures were recalibrated to a 0 to 100 scale, with each scale’s scoring aligned to the direction implied by its name [8]. The data collected, as detailed in Table 1 in the Appendix, provides a reasonable representation of the reference population in terms of key variables such as state and industry. To improve inferences about the reference population, a refined post-stratification weighting approach was employed [25]. This involved calculating weights for the nationally representative sample, based on gender, age, employment basis (full-time versus part-time), and sector type (public versus private), aligning the sample more closely with the actual workforce structure. Additional weights were calculated for separate analyses of occupational categories. This second set focused on gender and occupational categories but excluded employment basis and sector type, as these variables were instrumental in mapping the one-digit ANZSCO occupational categories, and consequently, the job classes. Weightings were verified through sensitivity analyses.

Overall, the representativeness of the results was enhanced through the combined use of quota sampling in data collection and weighting in analysis. This methodology adjusts for any overrepresentation or underrepresentation of specific worker groups within the sample, ensuring that the findings are reflective of the known population demographics [25]. The variable ‘control over working time’ was excluded from the analysis due to a high incidence of missing responses for two items in the measure, which resulted from formatting issues on the survey platform. Given the extent of missing data for the two items, imputation was deemed inappropriate, and the variable was removed from the analysis.

### Reliability analysis

Reliability analysis was conducted at two stages, prior to and after conducting Confirmatory Factor Analysis, by calculating Cronbach’s *α* for scales with 3 or more items and using Spearman-Brown Coefficient to assess inter-item correlations for two-item scales [26, 27].

### Exploratory Factor Analysis (EFA)

EFA was employed on the raw data to identify the underlying structure of the psychosocial work environment variables, aiming to uncover the latent dimensions within a large set of variables. This approach was informed by existing knowledge about the relationships from prior validations of COPSOQ [12, 28]. This aspect of the analysis provides a foundational understanding of the constructs, serving as a prelude to the construct validation analysis.

Prior to conducting the EFA, the suitability of the data for factor analysis was confirmed through preliminary checks. A Varimax rotation was performed to enhance the interpretability of the factors by maximising the variance of loadings within each factor, thereby simplifying the factor structure. The Kaiser–Meyer–Olkin (KMO) measure of sampling adequacy and Bartlett’s test of sphericity were used to assess the appropriateness of the data. The KMO index evalutes whether partial correlations among variables are small, while Bartlett’s test determines if the correlation matrix significantly differs from the identity matrix [29, 30]. The Eigenvalue criterion (≥ 1) was used to guide the selection of the number of factors, a standard method in EFA.

Following Lincke et al.’s [12], approach to the validation of the German version of COPSOQ III, EFA was conducted on two distinct elements of the dataset: ‘psychosocial work factors’ and ‘effects.’ The number of factors retained was determined using Kaiser’s criterion, which recommends retaining factors with Eigenvalues greater than one. This approach ensured a factor solution that balances both statistical robustness and interpretability. Principal axis factoring with a subsequent Varimax rotation was used, methods well-suited for analysing psychological data [29].

### Confirmatory factor analysis (CFA)

CFA was conducted on the individual raw items of each COPSOQ scale to confirm the construct validity of the scales. CFA allows for a direct hypothesis test of the factor structure, with each item hypothesised to load on a specific factor as defined by the COPSOQ’s conceptual model. The analysis was performed using maximum likelihood estimation within a structural equation modelling (SEM) framework, allowing for the systemic testing of the theorised structure of each scale by specifying items to load onto designated factors.

Model identification was ensured by applying appropriate constraints, and model fit was assessed using multiple goodness-of-fit indices to assess the adequacy of the factor structure. The Comparative Fit Index (CFI) and Tucker-Lewis Index (TLI) were used, with values close to or above 0.90 indicating acceptable fit. The Root Mean Square Error of Approximation (RMSEA), with a threshold of ≤ 0.05 for good fit and ≤ 0.08 for acceptable fit, was also considered, alongside the Standardised Root Mean Square Residual (SRMR), which should ideally be below 0.08 [31–33]. To enhance the robustness of the CFA results, cross-validation was performed on a randomly split sample, ensuring the stability of the factor structure [34].

### Inter-scale correlations

Bivariate Pearson correlation coefficients were calculated to evaluate the strength and direction of relationships between the scales used in this study. This analysis provided a comprehensive evaluation of psychosocial factors across a broad age range within the workforce. The Pearson correlation offers a measure of the degree of linear association between two continuous variables [35].

### Subgroup analyses

Subgroup analyses were performed to examine potential disparities in psychosocial workplace factors across demographics including gender, employment basis (full-time versus part-time), sector, and job class. These analyses aimed to identify differential impacts of workplace conditions on distinct employee groups, contributing to a deeper understanding of the psychosocial landscape within the national workforce. For ANZSCO categories, the study reported mean scores, standard deviations, and reliability coefficients, offering a comprehensive statistical profile of these occupational groups.

## Results

### Reliability analysis

Benchmarks for the measures of the COPSOQ III long version, specific to the Australian general workforce, along with the scale’s psychometric characteristics are presented in Tables 1 and 2. The internal consistency of the 31 scales, each comprising three or more items, was acceptable for all scales (< 0.7) except for Demands for Hiding Emotions (0.66). For scales with only two items, reliability was determined using the Spearman-Brown coefficient. Of the five two-item scales, one (meaning of work) demonstrated acceptable reliability (0.78), three scales (Predictability, role conflict and quality of work) demonstrated moderate reliability (> 0.6), and one scale (Variation of Work) had an unacceptably low value (0.24). Relatively low floor and ceiling effects were determined across most scales (see Initial RC in Tables 1 and 2).

**Table 1:** Australian population benchmarks ^a^ and reliability of scales for Australian general population (part 1)

Population Benchmarks							Scale Characteristics		
Measure	Desirable value	Mean	SD	No. of Items	Initial RC ^b^	RC post-CFA	Floor (%)	Ceiling (%)	Scale Missing (%)
**Quantitative Demands**	Low	40.7	21.3	4	0.78	0.78	4.1	0.5	0
**Work Pace**	Low	57.0	24.4	3	0.87	0.87	1.1	6.6	0
**Cognitive Demands**	High	59.9	21.2	4	0.76	0.76	0.5	3.9	0
**Emotional Demands**	Low	40.5	27.6	3	0.86	0.86	9.6	3.0	0
**Demands for Hiding Emotions**	Low	61.0	20.8	4	0.66	N/A^e^	0.8	3.4	0
**Influence**	High	51.0	22.5	6	0.85	0.85	1.4	2.1	0
**Possibilities for Development**	High	62.4	23.6	3	0.82	0.82	1.4	10.4	0
**Variation of Work**	High	45.6	20.4	2	0.24 ^c^	N/A ^d^	3.0	0.8	0
**Meaning of Work**	High	65.8	26.1	2	0.78 ^c^	0.78 ^c^	2.7	18.9	0
**Predictability**	High	58.3	24.7	2	0.63 ^c^	0.63 ^c^	3.2	7.7	0
**Recognition**	High	62.7	26.0	3	0.89	0.89	2.5	12.0	0
**Role Clarity**	High	72.7	20.8	3	0.83	0.83	0.8	14.6	0
**Role Conflict**	Low	42.7	26.4	2	0.65 ^c^	0.65 ^c^	8.2	3.7	0
**Quality of Leadership**	High	53.9	26.4	4	0.9	0.9	3.8	6.8	2.8
**Social Support from Internal Colleagues**	High	59.3	23.1	3	0.82	0.82	1.2	7.1	2.6
**Social Support from External Colleagues**	High	46.4	26.1	3	0.85	0.85	8.4	4.3	5.2
**Social Support from Colleagues**	High	52.9	22.0	6	0.87	0.87	0.9	3.5	5.6
**Social Support from Supervisors**	High	58.1	25.9	3	0.86	0.86	2.8	9.1	3.5
**Sense of Community at Work**	High	67.5	23.7	3	0.87	0.87	0.5	15.2	2.8
**Illegitimate Tasks **	Low	47.2	27.4	1	N/A^d^	N/A ^d^	9.8	8.0	0

^a^: Benchmarks calculated by means with SD and conflicts and offensive behaviours explained by frequencies (based on weighted data for gender, age, employment basis and employment sector); scale characteristics (number of items, reliability coefficient, floor, ceiling and scale missing percentages, based on unweighted data)); ^b^: Reliability Coefficient (RC) includes Cronbach’s *α* for scales with 3 or more items and Spearman-Brown Coefficient for two-item scales; ^c^: Spearman-Brown Coefficient significance; ^d^: Not applicable for single item scales;^e^: Not applicable due to the removal of the scale post-CFA

**Table 2:** Australian population benchmarks ^a^ and reliability of scales for Australian general population (part 2)

Population Benchmarks						Scale Characteristics			
Measure	﻿Desirable value	Mean	SD	No. of Items	Initial RC^b^	RC ^b^ post-CFA	Floor (%)	Ceiling (%)	Scale Missing (%)
**Job Insecurity**	Low	34.1	27.0	3	0.82	0.82	18.1	1.8	0
**Job Satisfaction**	High	67.0	19.9	5	0.85	0.85	0.3	4.9	0
**Work-Family Conflict (work-life imbalance)**	Low	40.1	26.7	5	0.91	0.91	5.4	1.7	0
**Quality of Work**	High	70.0	20.6	2	0.61 ^c^	0.61 ^c^	0.3	14.3	0
**Commitment to the Workplace**	High	57.2	23.8	5	0.86	0.86	1.2	4.0	0
**Work Engagement **	High	55.3	22.2	3	0.82	0.82	2.4	2.6	0
**Insecurity over Working Conditions **	Low	32.5	21.5	5	0.76	0.86	4.9	0.2	0
**Mutual Trust between Employees**	﻿High	63.8	22.5	3	0.70	0.75 ^c^	0.9	7.6	0
**Trust regarding Management**	High	61.9	21.9	4	0.77	0.77	0.9	5.7	0
**Organisational Justice**	High	59.8	24.1	4	0.90	0.90	1.8	7.1	0
**Self-rated Health**	High	57.0	23.7	1	N/A ^d^	N/A^d^	3.0	9.4	0
**Burnout**	Low	47.6	25.4	4	0.91	0.91	2.5	4.7	0
**Stress**	Low	40.2	25.6	3	0.88	0.88	7.6	2.5	0
**Sleeping Troubles**	Low	44.6	25.8	4	0.89	0.89	5.6	3.2	0
**Depressive Symptoms**	Low	34.7	26.1	4	0.89	0.89	10.8	1.4	0
**Somatic Stress**	Low	27.0	22.7	4	0.83	0.83	11.8	0.4	0
**Cognitive Stress**	Low	33.3	25.7	4	0.92	0.92	12.6	1.3	0
**Self-efficacy**	High	64.2	19.8	6	0.85	0.85	0.5	4.6	0
**Bullying** ^f^	Low	19.6%		1					0
**Threats of Violence** ^f^	Low	12.9%		1					0
**Physical Violence** ^f^	Low	10.2%		1					0
**Sexual Harassment** ^f^	Low	12.8%		1					0
**Conflicts & Quarrels** ^f^	Low	29.8%		1					0
**Gossip & Slander** ^f^	Low	33.6%		1					0
**Unpleasant Teasing** ^f^	Low	19.8%		1					0
**Cyber Bullying** ^f^	Low	9.7%		1					0

^a^: Benchmarks calculated by means with SD and conflicts and offensive behaviours explained by frequencies (based on weighted data for gender, age, employment basis and employment sector); scale characteristics (number of items, reliability coefficient, floor, ceiling and scale missing percentages, based on unweighted data)); ^b^: Reliability Coefficient (RC) includes Cronbach’s *α* for scales with 3 or more items and Spearman-Brown Coefficient for two-item scales; ^c^: Spearman-Brown Coefficient significance; ^d^: Not applicable for single item scales; ^f^: Proportion of sample experiencing offensive behaviours

### Exploratory factor analysis (EFA)

EFA was conducted on the two sets of factors related to ‘psychosocial working conditions’ and ‘effects’ [8] to uncover the underlying factor structure of the COPSOQ III scales administered to the Australia workforce (see Tables 4a&b in Appendix). The psychosocial working conditions set yielded a KMO value of 0.93 and a significant Bartlett’s Test (*χ*^*2*^(253) = 29,701.17, *p* < 0.001), while the effects factors set demonstrated a KMO value of 0.91 and a significant Bartlett’s Test (*χ*^*2*^(66) = 16,713.67, *p* < 0.001), confirming the appropriateness of factor analysis for both sets.

In conducting Principal Axis Factoring, the employment of Varimax rotation and considerations for interpretability guided the choice of a four-factor model pertinent to psychosocial working conditions. This model, encompassing 54% of the variance, provided a parsimonious and interpretable solution, with each factor demonstrating clear and meaningful loadings, thus aligning well with the theoretical constructs of the COPSOQ III, and a better balance between statistical adequacy and interpretability. The first factor explained approximately 26% of the variance, the second 13%, the third 8%, and the fourth 6%, cumulatively amounting to a substantial proportion of the total variance. In the Principal Axis Factoring of the second set on effects, Varimax rotation and interpretability led to a concise two-factor model explaining 57% of the variance in effects factors. The first factor accounted for 36% and the second for 21% of the variance, aligning with COPSOQ III’s theoretical constructs and ensuring clear factor interpretations. Self-efficacy from the first set and Self-rated General Health from the second set stood out.

The EFA summary tables, as indicated in Tables 4a and b in Appendix, demonstrates a four-factor solution with factor loadings of |0.40| or higher. The factor loadings indicate how each item correlates with the factors, providing insight into potential underlying constructs. The factors can be interpreted as follows:

- Psychosocial demands: This factor encompasses measures related to the pressures and challenges of the job, including 'Quantitative Demands', 'Work Pace', 'Cognitive Demands', 'Emotional Demands' and ‘Illegitimate Tasks’. These likely reflect the various psychological and emotional demands employees face in relation to their work.
- Interpersonal relations and employee resources: This factor comprises 'Meaning of Work', 'Predictability', 'Recognition', 'Role Clarity', 'Quality of Leadership', 'Social Support from Colleagues', 'Social Support from Supervisors', 'Sense of Community at Work', ‘Trust regarding Management’ and ‘Organisational Justice’. These measures appear to reflect the psychosocial and interpersonal aspects of work that can support employee health and wellbeing at work.
- Job security and trust: This factor comprises 'Job Insecurity', ‘Insecurity over Working Conditions’ and 'Mutual Trust between Employees'. These measures appear to relate to experiences of insecurity in relation to work.
- Work organisation and job content:This factor represents 'Influence', 'Possibilities for Development', and 'Variation', highlighting the empowerment and growth opportunities within a job. The measures reflect how much control employees have over their tasks, the availability of development opportunities, and the diversity in job content.
- Job satisfaction and engagement: This factor includes 'Job Satisfaction', 'Work Engagement', ‘Quality of Work’, and ‘Commitment to the Workplace’ highlighting the positive aspects of employees' psychological investment in their jobs and their overall contentment with their work conditions.
- Wellbeing and health outcomes: This factor includes Work-Family Conflict (work-life imbalance)’ and multiple measures of mental health including 'Burnout', 'Stress', 'Sleeping Troubles', 'Depressive Symptoms', 'Somatic Stress', and 'Cognitive Stress'. These measures are all closely related to the effects of work on health for workers in Australia.

## Confirmatory factor analysis (CFA)

The initial CFA of the COPSOQ III dataset with all of the COPSOQ items indicated a complex model with numerous parameters (845). The large sample size of 2228 observations (out of a total of 2446) provided robustness to the analysis. The Comparative Fit Index (CFI) of 0.897 and the Tucker-Lewis Index (TLI) of 0.886 suggest a reasonable fit, though they are slightly below the commonly accepted threshold of 0.9, indicating that a better fit could be obtained. The Root Mean Square Error of Approximation (RMSEA) of 0.036 (with a 90% confidence interval ranging from 0.035 to 0.036) and the Standardized Root Mean Square Residual (SRMR) of 0.067 were within acceptable limits, indicating a satisfactory fit of the model to the data.

The model’s complexity was also reflected in its information criteria values: Akaike Information Criterion (AIC) is 2,457,043.787, Bayesian Information Criterion (BIC) is 2,461,867.773, and Sample-size Adjusted Bayesian Information Criterion (SABIC) was 2,459,183.076. These values were high, as expected in complex models, and they balance the model’s goodness of fit with the number of parameters used. Overall, the initial CFA results suggest that the model was sufficiently complex to capture the underlying structure of the COPSOQ III dataset, although the fit indices were marginally below the ideal threshold. The high AIC, BIC, and SABIC values reflected the model’s complexity and number of parameters.

### Improving the model

In the process of refining the CFA model, specific items were selectively removed to improve model fit and construct validity, as detailed in Table 5 in Appendix. A total of seven items were removed, resulting in an improved fit and a more parsimonious factor structure [32, 33]. The items ‘Demands for Hiding Emotions’ – ‘he1’ and ‘he2’- were removed due to extreme high or low loadings indicating potential statistical anomalies. Other items, such as ‘Insecurity over Working Conditions’ – ‘iw5’, were excluded due to their low R-squared values which signified a weak relationship with the underlying factor. Additionally, items with high variances, such as ‘Demands for Hiding Emotions—he3’, were also removed, as they suggested poor fit and inconsistency with other items within the factor. The combined effect of these exclusions was a more robust and coherent factor structure, enhancing the overall interpretability and quality of the CFA model.

### Final CFA results

After the removal of items from the dataset, the Comparative Fit Index (CFI) and Tucker-Lewis Index (TLI) values were determined as 0.916 and 0.908 respectively, indicating a good fit of the model to the data, and suggesting that the latent variables are well represented by their respective indicators. The Root Mean Square Error of Approximation (RMSEA) value of 0.034, with a 90% confidence interval of 0.033 to 0.034 and a close fit probability of 1.000, further reinforced the model’s robustness.

The decision to exclude certain items was based on their poor factor loadings, R-squared values and high variances, which implied a weak contribution to their respective latent constructs. This strategic removal not only enhanced the overall model fit but also increased the explanatory power and reliability of the constructs within the model. The current results demonstrated stronger factor loadings and higher R-squared values for the retained items, indicating that they were significant and relevant representations of the underlying latent constructs (see Table 6 in Appendix).

The model’s improvement is partly due to higher R-squared values for many items, reflecting a larger proportion of variance explained by the latent constructs. This increase resulted from removing items that did not effectively contribute to explaining the constructs. The refined CFA model exhibited a sound statistical and theoretical structure, more accurately representing the constructs and aligning closely with the underlying theoretical framework.

In addition, a second set of reliability was performed post-CFA to show changes in reliability coefficients of the modified scales with two or more items in the final dataset. The result indicated improvements in reliability coefficients for ‘Insecurity over Working Conditions’ and ‘Mutual Trust between Employees’ (see Table 2).

## Correlations

The inter-scale correlations for the COPSOQ III variables illustrates the relationships between different psychosocial workplace factors (see Table 7 a, b and c in Appendix). For example, the positive correlation, between ‘Work Pace’ and ‘Cognitive Demands’ suggests that higher job complexity is associated with increased work speed. Social factors such as support from colleagues, are positively linked to leadership quality, highlighting the role of a supportive environment in leadership effectiveness.

Additionally, there is an inverse relationship between ‘Job Satisfaction’ and ‘Work-family Conflict’, indicating that higher job satisfaction is associated with lower levels of work-life imbalance and vice versa. Overly strong intercorrelations might suggest that scales do not measure distinct constructs [7]; however, only 8 out of 684 correlations exceeded 0.70, indicating that the constructs are generally distinct. The strongest observed correlation was between ‘Organisational Justice’ and ‘Trust in Management’ at 0.784. Other notable correlations included ‘Social Support from Supervisor’ and ‘Quality of Leadership’ at 0.725, and several high correlations involving stress: ‘Stress’ and ‘Burnout’ at 0.758, ‘Stress’ and ‘Depressive Symptoms’ at 0.766, and ‘Stress’ and ‘Cognitive Stress Symptoms’ at 0.712.

## Subgroup comparisons

### Gender and employment basis

The results, as presented in Table 8 in the Appendix, utilised independent samples t-tests to compare means between males and females, as well as between full-time and part-time employees across scale measures (e.g., quantitative demands, work pace). Notable findings include gender differences in work pace and emotional demands, with females reporting higher levels, and significant variations in cognitive demands and job influence based on full versus part-time work, with full-time employees reporting higher demands. Additionally, the table reports the prevalence of workplace violence like bullying and sexual harassment. For example, it shows that 24.1% of females experienced bullying, significantly higher than the 15.1% of males. Similarly, the rates of threats of violence were closely matched, with full-time employees reporting 12.9% compared to part-time employees at 12.8%.

### Job sector and class

To assess the variances in psychosocial workplace factors across different sectors and job classes, the study employed weighted data to conduct multiple comparisons. In Table 9 of the Appendix, scale measures were compared between public and private sectors, as well as between white-collar and blue-collar job classifications. Public sector employees generally experienced higher quantitative and emotional demands than their private sector counterparts. Similarly, white-collar workers reported higher cognitive demands compared to blue-collar workers. Effect sizes were quantified using Cohen’s* d*, indicating small to moderate effects. The results also extended to psychosocial adversities, with higher reports of bullying and threats of violence in the public sector and among white-collar employees. These findings suggest distinct psychosocial risk profiles inherent in different work environments and job types.

The tables 10a and 10b in Appendix present the means, standard variations of COPSOQ III scales and proportions of violence incidences for various ANZSCO occupational categories. It shows a detailed breakdown of psychosocial factors across diverse job roles. For example, professionals reported the highest emotional demands, with a mean score of 58.3. Meanwhile, managers showed relatively lower scores in emotional demands (42.9) but higher scores in influence (53.7) and possibilities for development (68.9), highlighting variations in psychosocial stressors and resources across job roles. High levels of work engagement and job satisfaction are noted across most roles, while certain categories indicate higher levels of conflict and stress, such as Professionals and Technicians. The tables further delineate the period prevalence of violence in the workplace over the past year, with percentages indicating that experiences of bullying, threats, physical violence, and other confrontational incidents are occupational hazards that differ markedly across various job categories.

## Discussion

Our validation of the international long version of the COPSOQ III in a representative sample of the Australian working population demonstrated that the instrument provides a diverse range of measures of the psychosocial work environment and worker health that are both valid and reliable in the Australian context. Most measures demonstrated satisfactory reliability coefficients, and the internal consistency reliability of the COPSOQ-III scales was determined to be satisfactory for the sample population as a whole. The majority of COPSOQ scales (33 out of 38) demonstrated good reliability, aligning with validation studies of COPSOQ-III in other countries [7, 8, 12]. The two-item scale, ‘Variation of Work’, demonstrated low reliability, as observed in other studies (e.g., Berthelsen et al., [7]). Additionally, one four-item scale (‘Demands for Hiding Emotions’) and three two-item scales (‘Predictability’, ‘Role Conflict’ and ‘Quality of Work’) exhibited moderate reliability within the sample population.

Analysis of the reliability of COPSOQ-III scales within the major occupational categories yielded similar results, with the 33 out of 38 of scales demonstrating good reliability across occupational categories The scale ‘Variation at Work’ had low reliability across occupational groups, indicating that in its present form the scale should not be used for the Australian working population.

Floor and ceiling effects were minor for all but two scales. The scale ‘Job Insecurity’ demonstrated a high floor effect, reflecting findings from prior studies into this measure [7, 8]. The scale ‘Meaning at Work’ demonstrated a high ceiling effect also reflecting prior studies of this measure [7, 8].

The EFA of COPSOQ III yielded a four-factor model for the factors related to psychosocial working conditions and a two-factor model for the effects factors. Given the complex and multifaceted nature of the psychosocial work environment and worker health, the models exhibited good fit. The selected models in the two-pronged factor approach aligned with Lincke et al. [8], captured the broad underlying structure of the COPSOQ scales, and provided a broad explanation of the associations between different COPSOQ measures. The derived models also provide an initial localised interpretation of its constructs within the Australian workforce. These factors—Psychosocial demands, Interpersonal relations and employee resources, job security and trust, Work organisation and job content, Job satisfaction and engagement and Mental health outcomes—largely correspond with the primary domains of the COPSOQ III and the factor structure identified in other national populations [8]. For example, Lincke et al. [8] determined seven factors within the German workforce: demands at work, Influence and development, interpersonal relations and leadership, work–individual interface, job insecurity, values at the workplace, and Health and well-being. These factors overlap significantly with the Australian model, highlighting the cross-national applicability of COPSOQ constructs while also reflecting contextual differences in the use of some measures and in how psychosocial dimensions manifest within national populations.

The higher-level groupings determined in this study, could support risk management by alerting researchers, assessors and practitioners to key psychosocial risk domains, including job demands, interpersonal relationships, job design and job insecurity as well as the psychosocial outcomes that employees experience as a result of these hazards, including satisfaction and engagement and mental health.

In the CFA analysis, the refinement of the factor model through the exclusion of seven items, including the entire four-item scale ‘demands for hiding emotions’, significantly enhanced the model’s fit and parsimony and indicated that the vast majority of COPSOQ measures are valid, and the items contribute meaningfully to their respective constructs. The removal of these items simplified the model and demonstrated enhanced factor loadings and elevated R-squared values for the remaining items.

Overall, the present study supports the reliability and construct validity of COPSOQ-III in Australia, providing a foundation for future research into the psychosocial work environment and valid psychosocial risk assessments in Australian workplaces that have national and international comparability. This study establishes that a wide range of COPSOQ III measures can be used to measure and assess the different dimensions of the psychosocial work environment and employee outcomes in Australia. This initial identification of the broad factor structure of COPSOQ-III in the Australian context provides a foundation for future refinements of the instrument, potentially revealing additional sub-factors.

The analysis of the COPSOQ III inter-scale correlations provides insights into the complex interrelationships between various psychosocial workplace factors. Berthelsen et al. [7] found that in a study using fewer COPSOQ scales, only 6 out of 378 correlations in the Swedish national sample were notable, with the strongest between Stress and Burnout. Similar to Berthelsen et al.’s findings [7], these results can inform future comparative analyses between individual and workplace levels in Australian studies where company data are available.

Further, the study’s exploration into the influences of gender, employment status, work sector and job class differences, including the distinction between various occupational categories, provides insights into important differences in experiences of the psychosocial work environment within the Australian workforce. These findings underscore the necessity of tailored approaches to workplace intervention and policy. For instance, the initial indication that there are distinct psychosocial hazard profiles across various occupational groups and demographics signals the need for targeted risk management strategies that prioritise different hazards in different work contexts. The benchmarks by occupational and demographic categories offer a valuable reference for workplace psychosocial hazard risk assessment and management, as well as for researchers seeking examine the psychosocial work environment in Australia.

### Strengths & limitations

Although the study is constrained by a relatively low response rate, our analysis provides no substantial evidence of selection bias impacting the reported benchmarks and mean scores for ANZSCO 1-digit major occupational categories. However, the study’s use of a paid panel sample might introduce bias due to the overrepresentation of certain demographic groups, such as higher socioeconomic respondents [36] and dominant racial/ethnic groups [37], which may not reflect the broader working population. This could skew the results towards the perspectives of these groups, potentially underrepresenting more marginalised members of the working population. Additionally, due to its cross-sectional nature, the study is limited in determining the directionality of associations between psychosocial work environment and, health and wellbeing outcomes. A further potential limitation of this study is that variation in organisation size may influence specific psychosocial risk factors. However, this was not explicitly accounted for in the weighting process and should be considered when applying the benchmarks to risk assessment in organisations. The smaller subgroup samples sizes may also limit the generalisability of the benchmarks. Subgroup results should be interpreted with caution. Future studies could target under-represented groups to improve representativeness. Finally, while we did not modify the phrasing of COPSOQ items in this study, future studies could investigate whether amending the wording enhances the relevance and applicability of COPSOQ III in Australia.

## Conclusion

Our assessment of COPSOQ III in an Australian working population sample confirmed the validity and reliability of the instrument. These results offer a foundation for future psychosocial risk assessments and research within Australian workplaces and provide a foundation for the future development of COPSOQ-III in Australia. The results provide working population benchmarks, as well as demonstrating the need for targeted intervention strategies that account for distinct work contexts and demographic profiles. Future development of COPSOQ in Australia should focus on adapting the instrument to the local context and identifying additional dimensions that are not currently covered. For example, given the unique challenges posed by geographic isolation in Australia, future iterations could incorporate measures of remoteness as a psychosocial risk factor. Additionally, sector-specific measures and benchmarks could be developed for high-risk industries such as healthcare, education, mining, and construction. To enhance the utility of COPSOQ III for practical psychosocial risk assessment, training and support are essential to ensure its effective implementation and analysis in the Australian context.

## Data Availability

Due to ethical constraints, the data supporting this study’s findings cannot be made publicly available. Access is contingent upon further approvals from the ethics committee and consent from funding institution.

## Appendix

**Table:** Key demographics of the sample compared to the Australian working population

		Sample (n)	Sample (%)	AU working population (%)	Chi-square *p*-value
**Gender****	Male	963	39.4	51.5	*p <* 0.001
	Female	1465	59.9	47.5	*p <* 0.001
	Other	18	0.7	1	*p =* 0.22 (NS: Not Significant)
**Age group*'****	18-29	122	13.1	24.1	*p <* 0.001
	30-39	392	36.1	24.3	*p <* 0.001
	40-49	1420	37.6	21.2	*p <* 0.001
	50-59	288	7.6	18.6	*p <* 0.001
	60-79	149	5.6	11.8	*p <* 0.001
**State****	ACT	63	2.6	1.8	*p =* 0.03
	NSW	677	27.7	31.2	*p =* 0.02
	QLD	492	20.1	20.4	*p =* 0.78 (NS)
	NT	12	0.5	1	*p =* 0.12 (NS)
	SA	202	8.3	6.8	*p =* 0.04
	TAS	74	3	2.1	*p =* 0.05
	VIC	660	27	25.9	*p =* 0.28 (NS)
	WA	266	10.9	10.6	*p =* 0.65 (NS)
**Occupational Main Division (Industry)****	Agriculture, Forestry and Fishing	75	3.1	2.2	*p =* 0.02
	Mining	58	2.4	2.1	*p =* 0.34 (NS)
	Manufacturing	178	7.3	6.3	*p =* 0.05
	Electricity, Gas, Water and Waste Services	56	2.3	1.2	*p <* 0.001
	Construction	105	4.3	9.5	*p <* 0.001
	Wholesale Trade	77	3.1	2.6	*p =* 0.12 (NS)
	Retail Trade	261	10.7	9.8	*p =* 0.05
	Accommodation and Food Services	164	6.7	6.8	*p =* 0.92 (NS)
	Transport, Postal and Warehousing	137	5.6	4.9	*p =* 0.23 (NS)
	Information Media and Telecommunications	96	3.9	1.4	*p <* 0.001
	Financial and Insurance Services	128	5.2	3.8	*p <* 0.001
	Rental, Hiring and Real Estate Services	92	3.8	1.6	*p <* 0.001
	Professional, Scientific and Technical Services	247	10.1	9	*p =* 0.08 (NS)
	Administrative and Support Services	105	4.3	3	*p <* 0.001
	Public Administration and Safety	40	1.6	6.4	*p <* 0.001
	Education and Training	221	9	8.4	*p =* 0.25 (NS)
	Health Care and Social Assistance	364	14.9	15.3	*p =* 0.67 (NS)
	Arts and Recreation Services	42	1.7	1.8	*p =* 0.84 (NS)
**Employment Basis****	Full-time	1888	77.2	68.5	*p <* 0.001
	Part-time/Casual	558	22.8	31.5	*p <* 0.001
**Sector Type****	Public (government units and units controlled by government)	644	26.3	17.3	*p <* 0.001
	Private (all other units)	1802	73.7	82.7	*p <* 0.001

*Participants’ Age Median: 40; Mean: 40.8, SD: 9.8

**Australian working population extracted from Australian Bureau of Statistics [18–22] databases

**Table:** Other demographic attributes of the sample

		n	Proportion (%)
**Geolocation**	Metropolitan	1746	71.4
	Inner-regional	364	14.9
	Outer-regional	238	9.7
	Remote	77	3.1
	Very remote	21	0.9
**Citizenship status/visa type**	Australian citizen	2187	89.4
	Australian Permanent Resident	192	7.8
	On a temporary visa with rights for full-time work	54	2.2
	On a temporary visa with rights for part-time work	13	0.5
**Origin**	Australian	2346	95.9
	Aboriginal or Torres Strait Islander heritage/origin	100	4.1
**Languages speaking **	Only English	1998	81.7
	More than one language	448	18.3
**Ethnic background**	Australian	1558	63.7
	Asian	358	14.6
	African	16	0.7
	European	234	9.6
	Latin American	25	1
	Middle Eastern	21	0.9
	Caucasian	117	4.8
	New Zealander	51	2.1
	North American	11	0.4
	Other	46	1.9
**Company/institution size**	200 or more persons	1061	43.4
	Between 20 to 200 persons	910	37.2
	Less than 20 persons	475	19.4
**Employment type**	Ongoing/Permanent	1892	77.4
	Fixed-term/Contract	224	9.2
	Casual	211	8.6
	Self-employed/Business owner	119	4.9

**Table:** Distribution of occupational categories and job classes in the sample vs. general Australian workforce

		Sample (n)	Sample (%)	AU working population (%)
**Occupational**** Category (ANZSCO one-digit)**	Managers	145	6.3	13.3
	Professionals	121	5.2	25.8
	Technicians and Trades Workers	90	3.9	13.8
	Clerical and Administrative Workers	758	32.8	12.8
	Community and Personal Service Workers	250	10.8	11.1
	Labourers	462	20	8.8
	Sales Workers	383	16.6	8
	Machinery Operators and Drivers	100	4.3	6.4
**Job Class**	White Collar	1364	59.1	69
	Blue Collar	945	40.9	31

Sources: Australian Bureau of Statistics [22, 23]

**Table:** EFA on psychosocial work factors: rotated factor matrix (unweighted data)

Measures	Factor loadings*			
	1	2	3	4
**Quantitative Demands**		0.54		
**Work Pace**		0.69		
**Cognitive Demands**		0.75		
**Emotional Demands**		0.71		
**Demands for Hiding Emotions**		0.62		
**Influence**	0.48			0.4
**Possibilities for Development**	0.5			0.6
**Variation**				0.6
**Meaning of Work**	0.45			0.43
**Predictability**	0.72			
**Recognition**	0.8			
**Role Clarity**	0.62			
**Role Conflict**		0.5	-0.58	
**Quality of Leadership**	0.78			
**Social Support from Colleagues**	0.69			
**Social Support from Supervisors**	0.77			
**Sense of Community at Work**	0.68			
**Illegitimate Tasks **		0.46	-0.45	
**Insecurity over Working Conditions **			-0.5	
**Mutual Trust between Employees**			0.59	
**Trust regarding Management**	0.68		0.42	
**Organisational Justice**	0.78			
**Self-efficacy**	*0.36***			

* Loadings ≥ |0.40| are shown; Eigenvalue ≥1; Total variance explained 54%; ** Highest loading

**Table:** EFA on effects: rotated factor matrix (unweighted data)

**Measures**	**Factor loadings***	
	1	2
**Job Satisfaction**		0.83
**Work Engagement **		0.53
**Quality of Work**		0.64
**Commitment to the Workplace**		0.81
**Work-Family Conflict (work-life imbalance)**	0.55	
**Burnout**	0.79	
**Stress**	0.85	
**Sleeping Troubles**	0.71	
**Depressive Symptoms**	0.83	
**Somatic Stress**	0.77	
**Cognitive Stress**	0.82	
**Self-rated Health**	-*0.33***	

* Loadings ≥ |0.40| are shown; Eigenvalue ≥1; Total variance explained 57%; ** Highest loading

**Table:** CFA model refinement - Item exclusion details

Item	Factor Loading	*R-squared*	Variance
Demands for Hiding Emotions - he1	1.000	0.037	532.595
Demands for Hiding Emotions - he2	5.511	0.639	352.184
Demands for Hiding Emotions - he3	2.346	0.165	571.805
Demands for Hiding Emotions - he4	4.888	0.554	396.268
Variation - va2	0.273	0.072	494.947
Insecurity over Working Conditions - iw5	0.100	0.007	722.106
Mutual Trust between Employees - te3	0.351	0.148	467.241

**Table:** CFA model refinement – Key measures in pre and post-modification analyses

Measure	Pre-Modification	Post-Modification	Ideal Benchmark
**Sample Size**	2228	2217	-
**Number of Parameters**	845	762	-
**CFI (Comparative Fit Index)**	0.897	0.916	≥ 0.9
**TLI (Tucker-Lewis Index)**	0.886	0.908	≥ 0.9
**RMSEA (Root Mean Square Error of Approximation)**	0.036	0.034	≤ 0.05
**SRMR (Standardized Root Mean Square Residual)**	0.067	0.050	≤ 0.08
**AIC (Akaike Information Criterion)**	2457043.787	2283241.960	Lower is better
**BIC (Bayesian Information Criterion)**	2461867.773	2287588.339	Lower is better
**SABIC (Sample-size adjusted BIC)**	2459183.076	2285167.349	Lower is better

**Table:** The inter-scale correlations the COPSOQ III on Australian data

	QD	WP	CD	ED	HE	IN	PD	VA	MW	PR	RE	CL	CO
**QD Quantitative demands**	--												
**WP Work pace**	.453**	--											
**CD Cognitive demands**	.417**	.580**	--										
**ED Emotional demands**	.477**	.466**	.538**	--									
**HE Hiding emotions**	.325**	.406**	.393**	.546**	--								
**IN Influence**	-.071**	-.060**	.138**	-.041*	-.170**	--							
**PD Possibilities for development**	-.045*	.060**	.292**	0.031	-.044*	.457**	--						
**VA Variation**	-0.007	-.062**	.173**	-0.032	-.146**	.333**	.456**	--					
**MW Meaning of work**	-.080**	.042*	.223**	.099**	-0.008	.307**	.555**	.355**	--				
**PR Job predictability**	-.260**	-.089**	-0.009	-.163**	-.197**	.472**	.482**	.260**	.429**	--			
**RE Job recognition**	-.282**	-.126**	-0.035	-.266**	-.248**	.464**	.507**	.286**	.427**	.692**	--		
**CL Role clarity**	-.309**	-.058**	-0.026	-.213**	-.136**	.287**	.425**	.151**	.418**	.578**	.630**	--	
**CO Role conflicts**	.422**	.381**	.388**	.462**	.382**	-0.005	-.044*	-.066**	-.053**	-.198**	-.281**	-.278**	--
**QL Quality of leadership**	-.230**	-.065**	.044*	-.165**	-.165**	.421**	.517**	.308**	.441**	.634**	.696**	.529**	-.187**
**SC Social support from colleagues**	-.136**	-0.006	.122**	-0.022	-.106**	.395**	.465**	.266**	.414**	.493**	.549**	.394**	-0.02
**SS Social support from supervisor**	-.196**	-.090**	0.023	-.181**	-.175**	.374**	.467**	.286**	.398**	.554**	.678**	.508**	-.205**
**SW Sense of Community at Work**	-.267**	-.080**	0.02	-.222**	-.178**	.309**	.430**	.220**	.398**	.495**	.610**	.568**	-.265**
**IT Illegitimate Tasks**	.454**	.317**	.343**	.433**	.373**	-.063**	-.107**	-.109**	-.152**	-.293**	-.338**	-.338**	.566**
**JI Job insecurity**	.202**	.161**	.084**	.181**	.156**	-.094**	-.175**	-.154**	-.209**	-.128**	-.214**	-.236**	.256**
**JS Job satisfaction**	-.336**	-.161**	-.046*	-.259**	-.263**	.399**	.522**	.320**	.480**	.591**	.661**	.563**	-.301**
**WF Work-family conflict**	.477**	.425**	.392**	.520**	.360**	-.051*	-.080**	-.088**	-.102**	-.207**	-.307**	-.304**	.569**
**QW Quality of work**	-.333**	-.104**	-0.017	-.215**	-.124**	.300**	.410**	.188**	.397**	.499**	.511**	.588**	-.272**
**CW Commitment to the workplace**	-.243**	-.098**	.055**	-.132**	-.201**	.395**	.566**	.352**	.651**	.611**	.678**	.528**	-.224**
**WE Work engagement**	-.182**	-0.019	0.037	-.152**	-.150**	.210**	.340**	.226**	.394**	.324**	.391**	.357**	-.256**
**IW Insecurity over working conditions**	.317**	.243**	.141**	.323**	.284**	-.171**	-.283**	-.217**	-.244**	-.291**	-.393**	-.396**	.419**
**TE Mutual trust between employees**	-.343**	-.246**	-.207**	-.377**	-.310**	.153**	.231**	.155**	.214**	.362**	.465**	.429**	-.524**
**TM Trust in management**	-.328**	-.167**	-.120**	-.298**	-.299**	.346**	.402**	.218**	.341**	.606**	.684**	.545**	-.428**
**JU Organisational justice**	-.308**	-.169**	-.091**	-.263**	-.281**	.428**	.449**	.272**	.363**	.649**	.743**	.534**	-.305**
**GH General health rating**	-.202**	-.093**	-.071**	-.144**	-.157**	.236**	.225**	.163**	.243**	.313**	.331**	.243**	-.061**
**BO Burnout**	.445**	.396**	.344**	.454**	.370**	-.154**	-.084**	-.179**	-.142**	-.262**	-.304**	-.215**	.397**
**ST Stress**	.401**	.342**	.298**	.408**	.353**	-.124**	-.111**	-.155**	-.154**	-.239**	-.283**	-.257**	.408**
**SL Troubles sleeping**	.299**	.273**	.253**	.358**	.302**	-.054**	-.089**	-.158**	-.125**	-.169**	-.230**	-.174**	.321**
**DS Depressive symptoms**	.353**	.283**	.218**	.381**	.310**	-.108**	-.132**	-.177**	-.193**	-.213**	-.284**	-.298**	.388**
**SO Somatic stress symptoms**	.337**	.290**	.251**	.395**	.262**	0.015	-0.034	-.120**	-.085**	-.126**	-.182**	-.256**	.385**
**CS Cognitive stress symptoms**	.376**	.294**	.250**	.380**	.292**	-.054**	-.090**	-.126**	-.154**	-.205**	-.238**	-.283**	.389**
**SE Self-efficacy**	-.141**	-0.011	.110**	-.079**	-.043*	.281**	.315**	.178**	.284**	.302**	.335**	.341**	-.045*

**Table:** The inter-scale correlations the COPSOQ III on Australian data

	QL	SC	SS	SW	IT	JI	JS	WF	QW	CW
**QL Quality of leadership**	--									
**SC Social support from colleagues**	.561**	--								
**SS Social support from supervisor**	.725**	.636**	--							
**SW Sense of Community at Work**	.591**	.584**	.634**	--						
**IT Illegitimate Tasks**	-.275**	-.114**	-.251**	-.271**	--					
**JI Job insecurity**	-.127**	-.152**	-.189**	-.273**	.222**	--				
**JS Job satisfaction**	.619**	.500**	.575**	.599**	-.353**	-.274**	--			
**WF Work-family conflict**	-.213**	-.094**	-.241**	-.320**	.460**	.332**	-.338**	--		
**QW Quality of work**	.480**	.381**	.459**	.546**	-.296**	-.226**	.606**	-.326**	--	
**CW Commitment to the workplace**	.625**	.533**	.570**	.571**	-.322**	-.219**	.682**	-.259**	.505**	--
**WE Work engagement**	.371**	.298**	.368**	.417**	-.285**	-.298**	.451**	-.301**	.361**	.498**
**IW Insecurity over working conditions**	-.299**	-.222**	-.324**	-.410**	.361**	.654**	-.457**	.478**	-.379**	-.357**
**TE Mutual trust between employees**	.384**	.255**	.389**	.531**	-.441**	-.349**	.436**	-.518**	.403**	.387**
**TM Trust in management**	.595**	.449**	.590**	.600**	-.415**	-.251**	.606**	-.379**	.541**	.579**
**JU Organisational justice**	.675**	.535**	.639**	.603**	-.361**	-.145**	.645**	-.284**	.510**	.626**
**GH General health rating**	.288**	.306**	.266**	.265**	-.098**	-.119**	.363**	-.162**	.244**	.328**
**BO Burnout**	-.231**	-.145**	-.238**	-.228**	.395**	.272**	-.356**	.551**	-.241**	-.279**
**ST Stress**	-.227**	-.127**	-.219**	-.261**	.390**	.354**	-.353**	.541**	-.267**	-.263**
**SL Troubles sleeping**	-.170**	-.100**	-.191**	-.204**	.308**	.285**	-.275**	.416**	-.195**	-.210**
**DS Depressive symptoms**	-.199**	-.126**	-.235**	-.294**	.370**	.406**	-.355**	.495**	-.300**	-.272**
**SO Somatic stress symptoms**	-.112**	-0.01	-.138**	-.241**	.341**	.381**	-.262**	.487**	-.234**	-.149**
**CS Cognitive stress symptoms**	-.149**	-.075**	-.176**	-.248**	.367**	.371**	-.321**	.494**	-.299**	-.224**
**SE Self-efficacy**	.290**	.290**	.284**	.335**	-.119**	-.231**	.369**	-.130**	.406**	.311**

*Pearson Correlation significant at the 0.05 level

**Pearson Correlation significant at the 0.01 level

**Table:** The inter-scale correlations the COPSOQ III on Australian data

	WE	IW	TE	TM	JU	GH	BO	ST	SL	DS	SO	CS
**WE Work engagement**	--											
**IW Insecurity over working conditions**	-.381**	--										
**TE Mutual trust between employees**	.358**	-.508**	--									
**TM Trust in management**	.389**	-.460**	.647**	--								
**JU Organisational justice**	.348**	-.359**	.499**	.784**	--							
**GH General health rating**	.184**	-.143**	.147**	.256**	.345**	--						
**BO Burnout**	-.250**	.360**	-.346**	-.300**	-.316**	-.423**	--					
**ST Stress**	-.277**	.415**	-.367**	-.305**	-.284**	-.357**	.758**	--				
**SL Troubles sleeping**	-.197**	.345**	-.304**	-.235**	-.216**	-.320**	.637**	.662**	--			
**DS Depressive symptoms**	-.315**	.450**	-.412**	-.310**	-.263**	-.351**	.682**	.766**	.587**	--		
**SO Somatic stress symptoms**	-.228**	.465**	-.388**	-.233**	-.156**	-.259**	.566**	.638**	.564**	.699**	--	
**CS Cognitive stress symptoms**	-.279**	.443**	-.388**	-.283**	-.229**	-.310**	.671**	.712**	.579**	.769**	.711**	--
**SE Self-efficacy**	.272**	-.248**	.196**	.288**	.307**	.299**	-.209**	-.229**	-.173**	-.304**	-.189**	-.257**

**Pearson Correlation significant at the 0.01 level

**Table:** Differences in psychosocial workplace factors over gender and employment status using t-tests and Cohen’s* d* for mean scales and Chi tests for percentage proportions (weighted data)

**Measure**	Gender				Employment basis			
	Male	Female	*p*	Cohen’s* d*	F-time	P-time	*p*	Cohen’s* d*
**Quantitative Demands**	40.3	41.0		0.0	43.1	36.4	***	0.3
**Work Pace**	54.7	59.4	***	-0.2	57.8	55.6	*	0.1
**Cognitive Demands**	58.6	60.9	**	-0.1	62.6	55.0	***	0.4
**Emotional Demands**	37.6	43.1	***	-0.2	42.0	37.8	***	0.2
**Demands for Hiding Emotions**	58.3	63.3	***	-0.2	61.1	60.8		0.0
**Influence**	53.1	48.8	***	0.2	53.2	46.9	***	0.3
**Possibilities for Development**	62.3	62.6		0.0	65.0	57.5	***	0.3
**Variation**	47.6	43.7	***	0.2	47.7	41.8	***	0.3
**Meaning of Work**	65.7	66.2		0.0	66.4	64.6		0.1
**Predictability**	60.4	56.5	***	0.2	58.2	58.4		0.0
**Recognition**	63.5	62.2		0.1	62.8	62.6		0.0
**Role Clarity**	73.1	72.7		0.0	71.4	75.0	***	-0.2
**Role Conflict**	42.7	42.7		0.0	46.0	36.8	***	0.4
**Quality of Leadership**	53.5	54.4		0.0	54.8	52.2	*	0.1
**Social Support from internal Colleagues**	57.9	60.9	**	-0.1	59.7	58.4		0.1
**Social Support from external Colleagues**	46.2	46.6		0.0	48.0	43.3	***	0.2
**Social Support from Colleagues**	52.1	53.9	*	-0.1	53.9	51.2	**	0.1
**Social Support from Supervisors**	58.2	58.5		0.0	59.5	55.5	***	0.2
**Sense of Community at Work**	66.3	68.9	*	-0.1	67.4	67.8		0.0
**Illegitimate Tasks **	45.6	48.3	*	-0.1	49.4	43.2	**	0.2
**Job Insecurity**	34.0	33.7		0.0	34.2	34.0		0.0
**Job Satisfaction**	68.1	66.2	*	0.1	66.9	67.1		0.0
**Work-Family Conflict (work-life imbalance)**	40.3	39.9		0.0	43.5	34.1	**	0.4
**Quality of Work**	70.2	70.0		0.0	69.3	71.1	*	-0.1
**Commitment to the Workplace**	57.4	57.3		0.0	58.2	55.3	**	0.1
**Work Engagement **	54.1	56.6	*	-0.1	55.0	55.8		0.0
**Insecurity over Working Conditions **	32.4	32.4		0.0	32.6	32.2		0.0
**Mutual Trust between Employees**	63.4	64.2		0.0	61.9	67.4	***	-0.2
**Trust regarding Management**	61.3	62.6		-0.1	60.6	64.2	***	-0.2
**Organisational Justice**	60.9	58.7	*	0.1	60.1	59.2		0.0
**Self-rated Health**	58.3	55.9	*	0.1	58.5	54.3	***	0.2
**Burnout**	42.7	52.1	***	-0.4	48.5	46.0	*	0.1
**Stress**	36.3	43.8	***	-0.3	41.0	38.9	*	0.1
**Sleeping Troubles**	41.1	47.9	***	-0.3	43.6	46.5	**	-0.1
**Depressive Symptoms**	31.1	37.8	***	-0.3	34.7	34.6		0.0
**Somatic Stress**	24.5	29.2	***	-0.2	27.6	26.1		0.1
**Cognitive Stress**	29.8	36.3	***	-0.3	33.9	32.4		0.1
**Self-efficacy**	65.3	63.2	**	0.1	64.7	63.3		0.1
Bullying	15.1%	24.1%	***		20.1%	18.7%		
Threats of Violence	11.9%	13.7%			12.9%	12.8%		
Physical Violence	9.0%	10.8%			10.4%	9.9%		
Sexual Harassment	11.0%	14.8%	**		14.1%	10.6%	*	
Conflicts & Quarrels	28.0%	30.1%			32.3%	25.3%	***	
Gossip & Slander	29.9%	37.2%	***		35.8%	29.6%	**	
Unpleasant Teasing	20.3%	19.1%			21.8%	16.1%	***	
Cyber Bullying	10.7%	9.1%			11.2%	7.2%	***	

*0.01≤ *p *<0.05

**0.001≤ *p* <0.01

****p*<0.001

**Table:** Differences in psychosocial workplace factors over sector and job class (weighted data)

Measure	Sector				Class			
	Public	Private	*p*	Cohen’s* d*	White Collar	Blue Collar	*p*	Cohen’s* d*
**Quantitative Demands**	46.4	39.6	***	0.3	44.4	39.8	***	0.2
**Work Pace**	60.9	56.3	***	0.2	59.3	57.5		0.1
**Cognitive Demands**	64.3	59.1	***	0.2	64.1	59.3	***	0.2
**Emotional Demands**	52.5	38.3	***	0.5	47.9	38.3	***	0.3
**Demands for Hiding Emotions**	67.8	59.7	***	0.4	63.5	60.5	**	0.1
**Influence**	47.7	51.6	**	-0.2	52.1	49.9	*	0.1
**Possibilities for Development**	64.8	61.9	*	0.1	66.7	61.0	***	0.3
**Variation**	47.2	45.3		0.1	48.0	44.9	**	0.2
**Meaning of Work**	69.1	65.2	**	0.2	69.4	64.4	***	0.2
**Predictability**	55.5	58.8	*	-0.1	57.5	57.1		0.0
**Recognition**	59.4	63.3	**	-0.2	61.2	62.7		-0.1
**Role Clarity**	69.2	73.3	***	-0.2	72.7	71.6		0.1
**Role Conflict**	51.1	41.2	***	0.4	47.0	43.3		0.1
**Quality of Leadership**	53.6	53.9		0.0	54.0	53.2		0.0
**Social Support from internal Colleagues**	58.9	59.3		0.0	60.7	57.8		0.1
**Social Support from external Colleagues**	48.9	45.9	*	0.1	49.2	44.6	***	0.2
**Social Support from Colleagues**	54.0	52.7		0.1	55.1	51.2	***	0.2
**Social Support from Supervisors**	56.7	58.4		-0.1	59.1	57.2		0.1
**Sense of Community at Work**	64.3	68.2	**	-0.2	67.3	66.6		0.0
**Illegitimate Tasks **	56.1	45.5	***	0.4	52.0	48.0	**	0.1
**Job Insecurity**	35.8	33.8		0.1	33.1	34.9		-0.1
**Job Satisfaction**	63.3	67.6	***	-0.2	66.1	66.7		0.0
**Work-Family Conflict (work-life imbalance)**	46.9	38.9	***	0.3	43.8	42.2		0.1
**Quality of Work**	64.1	71.1	***	-0.3	69.2	68.6		0.0
**Commitment to the Workplace**	57.6	57.1		0.0	58.2	57.6		0.0
**Work Engagement **	51.4	56.0	***	-0.2	54.5	54.8		0.0
**Insecurity over Working Conditions **	37.6	31.5	***	0.3	33.5	33.1		0.0
**Mutual Trust between Employees**	58.0	64.9	***	-0.3	61.6	62.6		0.0
**Trust regarding Management**	56.3	63.0	***	-0.3	59.8	60.8		0.0
**Organisational Justice**	55.0	60.7	***	-0.2	57.9	59.3		-0.1
**Self-rated Health**	56.8	57.0		0.0	57.7	56.8		0.0
**Burnout**	54.3	46.3	***	0.3	50.3	48.0		0.1
**Stress**	45.8	39.2	***	0.3	42.7	40.6		0.1
**Sleeping Troubles**	49.7	43.7	***	0.2	44.8	45.2		0.0
**Depressive Symptoms**	41.1	33.5	***	0.3	36.4	35.5		0.0
**Somatic Stress**	33.4	25.9	***	0.3	28.8	28.2		0.0
**Cognitive Stress**	41.1	31.9	***	0.4	34.8	34.7		0.0
**Self-efficacy**	60.2	65.0	***	-0.2	64.8	63.8		0.1
**Bullying**	32.9%	17.1%	***		25.2%	20.2%	*	
**Threats of Violence**	28.8%	9.9%	***		18.0%	13.1%	**	
**Physical Violence**	23.8%	7.2%	***		16.5%	9.3%	***	
**Sexual Harassment**	24.0%	10.7%	***		16.2%	12.4%	*	
**Conflicts & Quarrels**	39.7%	27.9%	***		36.2%	29.7%	**	
**Gossip & Slander**	43.0%	31.8%	***		38.1%	32.6%	*	
**Unpleasant Teasing**	28.1%	18.2%	***		22.2%	21.4%		
**Cyber Bullying**	19.7%	7.9%	***		13.6%	11.2%		

*0.01≤ *p* <0.05

**0.001≤ *p* <0.01

****p*<0.001

**Table:** Differences in psychosocial workplace factors across different occupational categories - ANZSCO (weighted data)

Measure	Managers			Professionals			Technicians and Trades Workers			Clerical and Administrative Workers		
	Mean	*SD*	RC*	Mean	*SD*	RC*	Mean	*SD*	RC*	Mean	*SD*	RC*
**Quantitative Demands**	41.8	*23.1*	0.83	50.0	*21.2*	0.82	45.7	*21.4*	0.77	45.0	*21.9*	0.80
**Work Pace**	54.2	*23.7*	0.84	62.4	*23.8*	0.87	61.5	*24.1*	0.86	56.9	*24.4*	0.88
**Cognitive Demands**	60.4	*19.9*	0.73	72.1	*19.3*	0.75	64.1	*23.2*	0.83	63.0	*20.6*	0.76
**Emotional Demands**	42.9	*27.5*	0.89	58.3	*29.5*	0.89	49.2	*27.7*	0.87	45.3	*27.9*	0.87
**Demands for Hiding Emotions**	62.7	*21.3*	0.68	68.9	*21.4*	0.75	60.5	*20.7*	0.70	63.3	*20.7*	0.66
**Influence**	53.7	*20.3*	0.84	50.7	*18.8*	0.80	55.8	*22.8*	0.86	50.6	*20.2*	0.83
**Possibilities for Development**	68.9	*20.8*	0.80	72.0	*19.7*	0.82	67.3	*19.1*	0.73	64.6	*22.8*	0.85
**Variation**	50.6	*18.4*	0.22**	53.3	*16.8*	0.08**	44.5	*17.4*	0.04**	48.1	*18.9*	0.24**
**Meaning of Work**	74.1	*22.6*	0.71**	76.6	*23.6*	0.83**	68.6	*23.7*	0.74**	68.5	*25.2*	0.8**
**Predictability**	57.1	*27.3*	0.72**	55.5	*23.7*	0.69**	60.0	*24.9*	071**	56.8	*23.4*	0.6**
**Recognition**	61.0	*25.8*	0.88	58.6	*24.7*	0.86	64.3	*23.4*	0.83	60.9	*24.7*	0.88
**Role Clarity**	75.1	*17.5*	0.75	73.5	*19.7*	0.87	70.9	*17.4*	0.75	71.2	*20.3*	0.84
**Role Conflict**	41.7	*26.2*	0.66**	51.8	*25.9*	0.66**	49.5	*26.9*	0.68**	45.3	*26.0*	0.64**
**Quality of Leadership**	53.8	*26.2*	0.91	52.2	*27.1*	0.92	57.2	*24.8*	0.87	54.0	*25.3*	0.89
**Social Support from internal Colleagues**	59.4	*21.8*	0.81	62.3	*19.0*	0.78	60.6	*23.2*	0.86	59.6	*22.4*	0.81
**Social Support from external Colleagues**	46.1	*25.1*	0.84	51.4	*23.8*	0.76	51.5	*24.5*	0.81	47.2	*25.6*	0.85
**Social Support from Colleagues**	52.9	*20.6*	0.85	56.9	*17.8*	0.78	56.3	*21.5*	0.87	53.5	*21.1*	0.85
**Social Support from Supervisors**	61.0	*25.1*	0.89	57.7	*24.8*	0.89	59.3	*25.8*	0.85	59.2	*23.9*	0.84
**Sense of Community at Work**	65.5	*25.0*	0.90	68.0	*22.1*	0.87	67.0	*23.7*	0.85	67.1	*23.0*	0.87
**Illegitimate Tasks **	47.6	*26.6*	N/A***	58.8	*24.2*	N/A***	55.1	*30.3*	N/A***	50.4	*25.5*	N/A***
**Job Insecurity**	33.3	*29.1*	0.86	29.4	*27.9*	0.87	35.5	*25.3*	0.78	33.0	*27.7*	0.83
**Job Satisfaction**	65.2	*21.9*	0.87	65.9	*18.1*	0.80	68.1	*18.1*	0.84	65.7	*18.8*	0.83
**Work-Family Conflict (work-life imbalance)**	41.2	*28.5*	0.93	47.7	*26.0*	0.91	49.2	*26.0*	0.89	41.1	*26.4*	0.91
**Quality of Work**	72.0	*20.3*	0.59**	69.4	*18.7*	0.64**	67.5	*19.8*	0.74**	68.8	*20.7*	0.58**
**Commitment to the Workplace**	58.4	*24.5*	0.85	59.7	*22.9*	0.87	61.2	*21.5*	0.84	57.0	*23.6*	0.86
**Work Engagement **	55.3	*22.8*	0.83	56.0	*21.9*	0.81	50.9	*22.8*	0.82	54.1	*21.4*	0.81
**Insecurity over Working Conditions **	31.9	*22.9*	0.80	33.0	*21.9*	0.80	36.4	*23.2*	0.81	32.2	*21.8*	0.75
**Mutual Trust between Employees**	62.0	*23.8*	0.73	60.8	*21.7*	0.71	59.8	*20.9*	0.64	63.5	*22.4*	0.72
**Trust regarding Management**	61.5	*21.6*	0.76	56.1	*20.1*	0.80	59.7	*18.4*	0.69	60.4	*21.2*	0.78
**Organisational Justice**	59.6	*24.4*	0.89	54.0	*23.7*	0.89	60.2	*22.6*	0.88	57.9	*23.0*	0.89
**Self-rated Health**	59.1	*22.6*	N/A***	58.3	*24.2*	N/A***	59.4	*22.3*	N/A***	57.2	*23.4*	N/A***
**Burnout**	43.8	*27.3*	0.94	54.1	*23.9*	0.89	55.5	*24.1*	0.91	50.0	*25.3*	0.91
**Stress**	36.3	*27.5*	0.91	45.8	*24.4*	0.87	47.2	*25.9*	0.88	41.6	*25.2*	0.87
**Sleeping Troubles**	40.0	*25.0*	0.92	44.3	*26.0*	0.90	49.8	*27.3*	0.91	45.0	*25.6*	0.89
**Depressive Symptoms**	30.0	*25.7*	0.90	35.5	*24.1*	0.87	44.9	*27.5*	0.90	33.6	*25.6*	0.89
**Somatic Stress**	24.4	*23.9*	0.88	28.4	*23.8*	0.83	35.7	*24.9*	0.84	26.1	*21.9*	0.81
**Cognitive Stress**	30.0	*27.3*	0.94	35.0	*24.5*	0.91	40.8	*26.3*	0.92	33.7	*25.5*	0.92
**Self-efficacy**	67.9	*19.4*	0.85	65.7	*17.6*	0.82	64.6	*19.0*	0.84	63.2	*19.1*	0.85
Bullying	22.1%			37.8%			20.7%			23.0%		
Threats of Violence	14.7%			27.1%			15.0%			14.4%		
Physical Violence	9.6%			26.9%			15.0%			11.7%		
Sexual Harassment	9.9%			20.4%			15.0%			14.0%		
Conflicts & Quarrels	27.1%			49.1%			38.6%			32.3%		
Gossip & Slander	29.3%			43.3%			43.6%			38.3%		
Unpleasant Teasing	17.6%			24.4%			28.5%			19.9%		
Cyber Bullying	12.5%			17.4%			18.8%			9.4%		

*Reliability Coefficients include Cronbach’s *α *for scales with 3 or more items and Spearman-Brown Coefficient for two-item scales

**Spearman-Brown Coefficient significance

***N/A: not applicable for single item scales

**Table:** Differences in psychosocial workplace factors across different occupational categories - ANZSCO (weighted data)

Measure	Community and Personal Service Workers			Labourers			Sales Workers			Machinery Operators and Drivers		
	Mean	*SD*	RC*	Mean	*SD*	RC*	Mean	*SD*	RC*	Mean	*SD*	RC*
**Quantitative Demands**	37.6	*21.6*	0.79	39.5	*21.2*	0.73	41.1	*18.9*	0.72	38.7	*19.1*	0.84
**Work Pace**	59.9	*24.1*	0.86	57.2	*24.5*	0.85	57.2	*23.6*	0.88	58.6	*23.1*	0.91
**Cognitive Demands**	57.7	*21.1*	0.73	56.8	*21.1*	0.74	60.6	*20.6*	0.75	61.9	*19.4*	0.79
**Emotional Demands**	40.0	*24.6*	0.83	38.4	*28.1*	0.86	38.5	*28.1*	0.87	37.6	*24.2*	0.80
**Demands for Hiding Emotions**	60.0	*21.1*	0.66	59.8	*20.6*	0.65	60.3	*20.4*	0.66	62.0	*19.6*	0.64
**Influence**	50.6	*22.4*	0.87	48.5	*23.5*	0.85	51.9	*23.0*	0.86	49.7	*23.1*	0.86
**Possibilities for Development**	58.9	*24.6*	0.82	58.0	*24.0*	0.81	64.5	*22.0*	0.78	61.8	*24.9*	0.86
**Variation**	41.8	*20.5*	0.29**	42.5	*20.6*	0.19**	47.0	*20.7*	0.34**	46.4	*20.1*	0.28**
**Meaning of Work**	57.3	*26.3*	0.76**	60.5	*26.1*	0.75**	69.8	*23.8*	0.76**	64.0	*26.1*	0.78**
**Predictability**	59.0	*24.0*	0.65**	57.4	*24.6*		58.1	*26.1*	0.7**	55.1	*23.8*	0.57**
**Recognition**	62.1	*26.2*	0.90	62.0	*26.7*	0.88	63.3	*26.7*	0.91	63.2	*26.7*	0.92
**Role Clarity**	72.2	*20.5*	0.83	70.7	*21.7*	0.83	73.0	*22.2*	0.85	71.3	*22.4*	0.85
**Role Conflict**	44.8	*25.6*	0.64**	42.2	*26.2*	0.55**	42.6	*27.2*	0.61**	46.2	*24.3*	0.71**
**Quality of Leadership**	53.9	*24.3*	0.89	51.8	*26.1*	0.89	54.2	*27.8*	0.92	54.4	*27.4*	0.91
**Social Support from internal Colleagues**	60.6	*21.0*	0.82	56.5	*24.1*	0.82	58.7	*24.0*	0.83	58.9	*23.0*	0.85
**Social Support from external Colleagues**	48.5	*23.6*	0.82	45.2	*26.9*	0.86	44.5	*26.4*	0.88	43.7	*28.0*	0.91
**Social Support from Colleagues**	54.7	*19.5*	0.86	51.0	*23.1*	0.88	51.8	*22.7*	0.87	50.8	*23.8*	0.91
**Social Support from Supervisors**	58.9	*25.5*	0.85	54.3	*27.0*	0.88	59.2	*25.6*	0.87	59.4	*26.8*	0.89
**Sense of Community at Work**	68.1	*23.3*	0.85	65.0	*23.9*	0.85	67.8	*24.6*	0.90	67.9	*20.9*	0.88
**Illegitimate Tasks **	45.7	*28.3*	N/A***	47.6	*28.9*	N/A***	46.0	*26.6*	N/A***	51.1	*23.0*	N/A***
**Job Insecurity**	36.0	*26.0*	0.78	36.8	*25.9*	0.79	34.5	*26.5*	0.80	32.1	*25.8*	0.78
**Job Satisfaction**	65.9	*20.3*	0.89	65.1	*19.6*	0.84	67.5	*20.8*	0.86	68.4	*22.0*	0.86
**Work-Family Conflict (work-life imbalance)**	38.3	*26.6*	0.91	42.0	*25.8*	0.89	40.8	*26.6*	0.91	44.4	*27.2*	0.92
**Quality of Work**	68.2	*19.1*	0.61**	68.4	*20.9*	0.68**	70.6	*20.6*	0.65**	66.4	*22.1*	0.63**
**Commitment to the Workplace**	54.5	*24.1*	0.87	55.0	*23.5*	0.83	58.5	*23.0*	0.86	60.9	*24.3*	0.89
**Work Engagement **	55.2	*22.5*	0.82	53.4	*21.8*	0.79	54.9	*22.7*	0.83	57.4	*20.7*	0.83
**Insecurity over Working Conditions **	34.3	*19.9*	0.70	35.7	*21.4*	0.74	31.9	*21.1*	0.77	30.0	*20.6*	0.72
**Mutual Trust between Employees**	62.3	*21.8*	0.67	62.5	*21.3*	0.66	63.2	*23.4*	0.74	61.8	*22.2*	0.71
**Trust regarding Management**	62.5	*21.1*	0.73	60.7	*21.6*	0.76	61.4	*23.5*	0.82	60.0	*22.5*	0.81
**Organisational Justice**	59.5	*24.1*	0.91	58.9	*24.0*	0.89	59.6	*24.5*	0.91	59.6	*23.8*	0.92
**Self-rated Health**	54.7	*21.7*	N/A***	57.4	*25.1*	N/A***	56.8	*24.1*	N/A***	56.0	*23.9*	N/A***
**Burnout**	47.1	*26.6*	0.92	49.3	*24.2*	0.90	46.7	*24.9*	0.92	47.5	*26.2*	0.92
**Stress**	41.2	*25.2*	0.86	41.8	*24.7*	0.88	39.3	*25.6*	0.88	40.1	*27.5*	0.92
**Sleeping Troubles**	45.1	*23.1*	0.86	46.3	*25.4*	0.88	43.7	*26.4*	0.88	45.1	*26.3*	0.91
**Depressive Symptoms**	38.0	*26.6*	0.89	38.7	*25.4*	0.88	32.6	*25.1*	0.88	33.7	*27.2*	0.90
**Somatic Stress**	29.5	*23.4*	0.81	29.6	*23.4*	0.83	27.2	*22.6*	0.83	27.0	*21.4*	0.80
**Cognitive Stress**	34.8	*25.8*	0.92	36.8	*25.4*	0.91	32.7	*25.8*	0.93	33.5	*26.2*	0.93
**Self-efficacy**	62.6	*19.4*	0.83	62.8	*19.7*	0.85	64.2	*20.5*	0.88	64.9	*17.5*	0.87
Bullying	17.3%			20.3%			19.1%			21.3%		
Threats of Violence	14.4%			17.6%			10.1%			9.2%		
Physical Violence	13.8%			13.1%			6.4%			7.0%		
Sexual Harassment	19.0%			16.3%			11.7%			7.1%		
Conflicts & Quarrels	28.8%			34.3%			26.1%			26.8%		
Gossip & Slander	34.1%			35.9%			28.7%			32.4%		
Unpleasant Teasing	20.0%			24.3%			18.6%			20.6%		
Cyber Bullying	8.2%			15.1%			8.0%			8.5%		

*Reliability Coefficients include Cronbach’s *α *for scales with 3 or more items and Spearman-Brown Coefficient for two-item scales

**Spearman-Brown Coefficient significance

***N/A: not applicable for single item scales

## Abbreviations

ABS: Australian Bureau of Statistics
AIC: Akaike Information Criterion
ANZSCO: Australian and New Zealand Standard Classification of Occupations
BIC: Bayesian Information Criterion
SABIC: Sample-size Adjusted Bayesian Information Criterion
CFA: Confirmatory Factor Analysis
CFI: Comparative Fit Index
COPSOQ: Copenhagen Psychosocial Questionnaire
EFA: Exploratory Factor Analysis
KMO: Kaiser–Meyer–Olkin
OHS: Occupational Health and Safety
RMSEA: Root Mean Square Error of Approximation
SD: Standard Deviation
TLI: Tucker-Lewis Index
